# SARS-CoV2 Infection During Pregnancy Causes Persistent Immune Abnormalities in Women Without Affecting the Newborns

**DOI:** 10.3389/fimmu.2022.947549

**Published:** 2022-07-14

**Authors:** Elena Vazquez-Alejo, Laura Tarancon-Diez, Itzíar Carrasco, Sara Vigil-Vázquez, Mar Muñoz-Chapuli, Elena Rincón-López, Jesús Saavedra-Lozano, Mar Santos-Sebastián, David Aguilera-Alonso, Alicia Hernanz-Lobo, Begoña Santiago-García, Juan Antonio de León-Luis, Patricia Muñoz, Manuel Sánchez-Luna, María Luisa Navarro, Mª Ángeles Muñoz-Fernández

**Affiliations:** ^1^ Immunology Section, Laboratory of ImmunoBiology Molecular, Hospital General Universitario Gregorio Marañón (HGUGM), HIV-HGM BioBank, Madrid, Spain; ^2^ Infectious Diseases in Paediatric Population, Gregorio Marañón Research Institute (IiSGM) and University Hospital, Madrid, Spain; ^3^ Infectious Diseases Section, Department of Paediatrics, Hospital General Universitario Gregorio Marañón (HGUGM), Madrid, Spain; ^4^ Department of Obstetrics and Gynecology, Hospital General Universitario Gregorio Marañón (HGUGM), Madrid, Spain; ^5^ Department of Neonatology, Hospital General Universitario Gregorio Marañón (HGUGM), Madrid, Spain; ^6^ Department of Clinical Microbiology and Infectious Diseases, Hospital General Universitario Gregorio Marañón (HGUGM), CIBER Enfermedades Respiratorias (CIBERES), Madrid, Spain; ^7^ Faculty of Medicine, Universidad Complutense de Madrid, Madrid, Spain; ^8^ CIBER of Infectious Diseases (CIBERINFEC), Madrid, Spain

**Keywords:** SARS-CoV2, pregnancy, SARS-CoV2 exposed newborns, immune system, longitudinal analysis

## Abstract

SARS-CoV2 infection in pregnancy and exposed newborns is poorly known. We performed a longitudinal analysis of immune system and determined soluble cytokine levels in pregnant women infected with SARS-CoV2 and in their newborns. Women with confirmed SARS-CoV2 infection and their exposed uninfected newborns were recruited from Hospital General Universitario Gregorio Marañón. Peripheral blood mononuclear cells (PBMCs), cord cells and plasma were collected at birth and 6 months later. Immunophenotyping of natural killer (NK), monocytes and CD4/CD8 T-cells were studied in cryopreserved PBMCs and cord cells by multiparametric flow cytometry. Up to 4 soluble pro/anti-inflammatory cytokines were assessed in plasma/cord plasma by ELISA assay. SARS-CoV2-infected mothers and their newborns were compared to matched healthy non-SARS-CoV2-infected mothers and their newborns. The TNFα and IL-10 levels of infected mothers were higher at baseline than those of healthy controls. Infected mothers showed increased NK cells activation and reduced expression of maturation markers that reverted after 6 months. They also had high levels of Central Memory and low Effector Memory CD4-T cell subsets. Additionally, the increased CD4- and CD8-T cell activation (CD154 and CD38) and exhaustion (TIM3/TIGIT) levels at baseline compared to controls remained elevated after 6 months. Regarding Treg cells, the levels were lower at infected mothers at baseline but reverted after 6 months. No newborn was infected at birth. The lower levels of monocytes, NK and CD4-T cells observed at SARS-CoV2-exposed newborns compared to unexposed controls significantly increased 6 months later. In conclusion, SARS-CoV2 infection during pregnancy shows differences in immunological components that could lead newborns to future clinical implications after birth. However, SARS-CoV2 exposed 6-months-old newborns showed no immune misbalance, whereas the infected mothers maintain increased activation and exhaustion levels in T-cells after 6 months.

## Introduction

In December 2019, an outbreak of a disease called pneumonia of unknown cause emerged in Wuhan, Hubei Province (China) (1). The causative agent of these pathologies was identified quickly by different laboratories as a novel coronavirus. A few weeks after the outbreak, the spread of this disease from that Chinese city has become a Public Health Emergency of International Concern, designated by World Health Organization (WHO) (2). At first, this virus was named “2019 novel coronavirus” (2019-nCoV). Later it was defined as the cause of the disease: “Severe Acute Respiratory Syndrome Corona Virus-2” (SARS-CoV2) and finally, has been termed CoVid-19 by WHO on February 11, 2020 (3). Thus far, the pandemic of SARS-Cov-2 has registered around 535 million cases and 6,31 million deaths around the world by June 2022.

Most SARS-CoV2 infected patients show moderate symptoms, but approximately 15% of them progress to severe pneumonia and other serious pathologies requiring hospitalization that can trigger death (4). The severity of the infection in adults is associated with certain risk factors such as age, sex and ethnicity, lifestyle habits and underlying comorbidities (5–7). Pregnant women have greater risk of suffering from severe CoVid-19 (8) and the general pregnancy complications like maternal age, diabetes, obesity or hypertension are considered risk factors of SARS-CoV2 infection morbidity (9).

The unique immune status with countless physiological and immunological changes during pregnancy including greater oxygen consumption, due to a decrease in functional residual capacity and less compliance of the rib cage, as well as alterations in the count of T lymphocytes, responsible for the adaptive immune response, make them especially vulnerable to virological and bacteriological agents that cause respiratory diseases and severe pneumonia, which may result in higher maternal and fetal morbidity and mortality (10). Additionally, the overexpression in placenta and fetal organs of the angiotensin-converting enzyme 2 (ACE2) receptor (11), that mediates SARS-CoV2 entry, is also involved in triggering an anti-inflammatory, anti-thrombotic and vasodilatory response that promotes the development of the fetus (12), and may provide favourable conditions for SARS‐CoV2 infection during pregnancy.

SARS-CoV2 infection in pregnant women is characterized by the similar symptoms as in general population: fever, cough, muscle pain, gastrointestinal symptoms and dyspnea (13). Only a small fraction of pregnant patients developed severe disease, and mortality from COVID-19 was rarely reported. However, a high proportion of preterm births, pre-eclampsia and cesarean delivery has been reported in those women (14).

Data concerning the immunological status and inflammatory profile of infected women and their exposed newborns are scarce. The inflammation caused by SARS-CoV2 can lead to alterations in components of innate and adaptive immune system during pregnancy and may also affect immunological newborns development (15), as occurs with the presence of the Human Immunodeficiency Virus (HIV) or the Hepatitis C Virus (HCV) in pregnant women, with or without vertical transmission (16, 17).

The probability of viral vertical and perinatal transmission is still controversial. Some studies support that transmission of SARS-CoV2 during pregnancy is extremely low (18–20) while other published series have reported several cases of CoVid-19 infected newborns (21–24). Nevertheless, it has been observed that a potent antibody response (IgM, IgG and IgA, especially) occurs in the serum and milk of mothers infected with SARS-CoV2 during pregnancy that can be transferred to the foetus before and after the birth. This points toward a possible protection of newborns from future infections (25). Despite no conclusion on vertical transmission, it is still unclear if the infection of pregnant women may have a significant impact on the fetal immune system, structure and function development of the foetus and newborns due to the inflammatory response against the virus.

The objective of this study was to perform a longitudinal study of the immune profiles of SARS-CoV2 infected mothers during pregnancy and their newborns at birth and at 6 months, in comparison to uninfected mothers and unexposed newborns.

## Materials and Methods

### Study Participants and Design

Pregnant women and their newborns at the Hospital General Universitario Gregorio Marañón (HGUGM) from Madrid (Spain) between March-November 2020 were recruited following these inclusion criteria: 1) women with positive SARS-CoV2 RT-PCR or SARS-CoV2 anti-IgG during pregnancy or childbirth (n=29); 2) inclusion of their newborns (n=25) and 3) mothers and newborns whose plasma and PBMCs were available in the Spanish HIV HGM BioBank. The studied group was referred to as SARS-CoV2 MOTHERS’ group (SCV2-M) (n=29) and they and their exposed newborns at childbirth were compared with a reference group of healthy pregnant non-SARS-CoV2 infected mothers’ group (previous negative SARS-CoV2 RT-PCR neither SARS-CoV2 anti-IgM/IgG) matched by age, called UNINFECTED MOTHERS’ group (UM) and their non-exposed newborns (n=16). Six months later, a longitudinal study of some of those SARS-CoV2 infected mothers (n=15) and their exposed newborns (n=12) were also performed.

The study was approved by the Ethics Committee of HGUGM (Ref: IRB 0000605). Informed consent was obtained from all the mothers and newborns’ legal guardians before the inclusion in the cohort GESNEO-COVID.

### Clinical Data and Laboratory Determinations

Clinical and epidemiological data of mothers and their newborns were collected from the hospital’s medical history through Research Electronic Data Capture (REDCap) platform, hosted on a server at the Instituto de Investigación Sanitaria Gregorio Marañón (IiSGM), Madrid (Spain). PBMCs, plasma and associated clinical data were provided by the Spanish HIV-HGM BioBank and HGUGM Paediatric Infectology Department, respectively.

Mothers were examined for the presence of SARS-CoV2 by PCR in respiratory samples (nasopharyngeal swabs) or by detecting IgG antibodies in maternal serum by SARS-CoV2 IgG II Quant Reagent Kit (Abbott, Chicago, USA) during childbirth. Newborns were tested for SARS-CoV2 infection (nasopharyngeal PCR and SARS-CoV2 anti-IgG in serum samples by SARS-CoV2 IgG II Quant Reagent Kit) at birth and 15 days later. Additionally, thirty milliliter samples of fresh whole blood from pregnant women (SARS-CoV2 and UM groups) and ten milliliter samples of fresh cord blood from newborns were collected in ethylene diamine tetra-acetic acid tubes at childbirths. Similarly, total fresh blood was also collected from SARS-CoV2 mothers and newborns 6 months after. Plasma and PBMCs were immediately isolated by Ficoll-Paque density gradient centrifugation and stored at -20°C and -170°C in the Spanish HIV- HGM BioBank respectively, until their use.

Proinflammatory (IL-6, TNFα and IL-17) and anti-inflammatory (IL-10) cytokine levels were quantified in plasma and cord plasma samples at birth and six months later in all groups by ELISA assay (R&D Systems, Minneapolis, MN) according to the provided manufacturer’s instructions. All samples were measured in duplicate.

### Cell Immunophenotyping

Immunophenotyping of immune components (T lymphocytes, natural Killer [NK] and monocytes) were performed using multiparametric flow cytometry following that hierarchy order according to sample availability. Due to this limitation, the number of patients included in each sub-analysis during the development of this study has varied.

Briefly, for all cytometry panels in mothers’ samples, PBMCs were thawed, washed with phosphate buffered saline (PBS) containing 3% of Bovine Serum Albumin (BSA) (Millipore) and stained for 30 min with surface antibodies markers, including cell viability LIVE/DEAD fixable Aqua Blue Dead Cell Stain (Life Technologies, CA, USA) and markers for linage, CD56, CD19 and CD14, CD3 and CD4 or CD8; maturation, CD45RA and CD27; activation, HLA-DR, CD154, CD137 and CD38; marker for recent thymic emigrants, CD31; senescence, CD57; exhaustion markers, TIM-3, PD1, LAG-3 and TIGIT and IL-7 (CD127) and IL-2 receptors (CD25). Additionally, a subset of PBMCs was permeabilized with eBioScience FoxP3/Transcription Factor Staining Buffer Set (Thermo Fisher Scientific, Massachusetts, USA) and stained with the intranuclear transcription marker FoxP3-PE for T regulatory cells (Treg) identification. All of them were distributed into three different T-cell cytometry panels. Lymphocytes were defined as viable cells having low forward/side scatter and expressing CD3, and/or CD8/CD4, but not CD19, CD14 and CD56. Detailed information concerning the panels’ designs can be shown at **Supplementary Table 1**. The T-cell maturation subsets defined based on the expression of CD45RA and CD27 as naïve (CD45RA+CD27+), central memory (CM; CD45RA−CD27+), effector memory (EM; CD45RA−CD27−) and terminally differentiated (TemRA; CD45RA+CD27−). Treg were defined by CD4, FoxP3 and CD25 expression. A representative gating strategy for T cells can be found in **Supplementary Figure 1A**. Isotypes controls were included for CD154, CD137, TIM-3, LAG-3, TIGIT, PD-1, CD31, FoxP3 and CD25.

For NK-cells immunophenotyping, cells were stained with LIVE/DEAD fixable Aqua Blue Dead Cell Stain for viability; lineage, CD56, CD16, CD3, CD14 and CD19; maturation, CD57 and TIM-3; activation, HLA-DR and CD158b; C-type lectin-like activating NKG2D and inhibiting NKG2A receptor markers for NK-cell phenotype (detailed list of antibodies at **Supplementary Table 1**). To identify NK cell, viable cells, negative for CD3, CD14 and CD19 were classified in three subsets according to the expression of CD56 and CD16: CD56^high^, CD56^dim^ (that includes CD16^high^ subset) and CD56^neg^ subsets. Schematic gating strategies for NK cells can be found in **Supplementary Figure 1B**. Isotype controls were included for TIM-3, NKG2D, NKG2A and CD158b.

For monocyte immunophenotyping (detailed list of antibodies at **Supplementary Table 1**), cells were stained with surface markers LIVE/DEAD fixable Aqua Blue Dead Cell Stain for viability; CD14, CD16, HLA-DR, CD56, CD19, CD3 for linage and monocytes’ subsets identification; cell adhesion markers, CD62L and CD49d; activation, CD163 and CD40.Then cells were permeabilized with BD Cytofix/Cytoperm (BD Biosciences) and stained with CD287 (Toll-like receptor 7, [TLR7]) for single RNA strain receptor. Monocytes were defined as viable cells negative for CD56, CD19 and CD3 and positive for HLA-DR and CD14. Schematic gating strategies for monocytes can be found in **Supplementary Figure 1C**. Isotype controls were included for CD163, CD62L, CD40, CD49d and TLR7.

In newborns, cord and blood PBMCs were thawed, washed and stained using three simplified flow cytometry panels described above including cell viability, CD8 T and CD4 T cells distribution, CD45RA and CD31 for naïve and recent thymic emigrant T cells and NK and monocyte subsets distribution. For this analysis, the frequency of every cell subset was quantified respect the total live PMBCs.

Acquisition was carried out in a Gallios flow cytometer (Beckman Coulter). Before acquisition, cells were fixed with 4% paraformaldehyde (PFA). At least 1 million events were acquired for each condition. FlowJo V10 software (BD Biosciences) was used for data analysis.

### Statistical Analysis

Continuous variables were expressed as medians and interquartile ranges (IQR). Categorical variables were expressed as number and percentages. Differences between categorical and continuous values were determined using chi-square test and Mann Whitney *U*-test respectively. Wilcoxon matched-pairs signed-rank test was conducted to compare evaluation time for each group since childbirth to 6 months later. Correlations were assessed using Spearman’s rank test. P-values <0.05 were considered statistically significant. The Statistical Package for the Social Sciences software (SPSS 20.0, Chicago, IL, USA) was used for the statistical analysis. Graphs were generated using GraphPad Prism 9.0 (GraphPad Software, Inc., San Diego, CA, USA).

## Results

### Characteristics of Studied Subjects

General and clinical characteristics of the 29 SARS-CoV2 infected mothers were enrolled in **Table 1**. Of them, 26 had a positive SARS-CoV2 RT-PCR and 8 SARS-CoV2 anti-IgG during pregnancy or childbirth. Only 14 had symptoms, becoming severe (shortness of breath, chest pain, aphasia, pneumonia) in 4 of them, but none required admission to the Intensive Care Unit (ICU). General and clinical data from SARS-CoV2 infected mothers’ newborns (n=25) can be shown in **Table 2**. They had a median gestational age of 39.4 weeks, 15 of them born vaginally (eutocic + instrumental) and 16 were breastfed at hospital discharge. Of 25 exposed uninfected children, although all of them had negative SARS-CoV2 RT-PCR at birth, 8 developed symptoms, being severe (respiratory distress syndrome, apnea break) in 4 of them; 5 were admitted to the Neonatal Intensive Care Unit (NICU) for prematurity, and 3 presented a positive SARS-CoV2 anti-IgG at birth of maternal origin. In general, health status and anthropometric measurements were within normal ranges according to gestational age.

**Table 1:** Infected SARS-CoV2 mothers’ characteristics.

Parameters	Values
**CoVid Mother – n**	29
**Age (years)**	31.4 [19.1-41.6]
**Ethnicity – n (%)**	
**White/Caucasian**	17 (58.6)
**Latin American**	11 (38)
**Arab**	1 (3.4)
**Comorbidities – n (%)**	
**Obesity**	1 (3.4)
**Hypertension**	2 (6.9)
**Diabetes**	1 (3.4)
**Other**	4 (13.8)
**SARS-CoV2 PCR (+) – n (%)**	26 (89.7)
**Pregnancy**	10 (27)
**Childbirth**	16 (59.3)
**SARS-CoV2 Serology (+) – n (%)**	
**Pregnancy IgM (+)**	1 (3.4)
**Pregnancy IgG (+)**	8 (27.6)
**Gestational age at diagnosis (weeks)**	38.1 [19.4-40.9]
**Symptoms – n (%)**	14 (48.3)
**Severe symptoms**	4 (13.8)
**Mild symptoms**	10 (34.5)
**Time of symptoms (days)**	6.5 [1.0-14.0]
**Required Hospitalization**	7 (24.1)
**Time of hospitalization (days)**	3.0 [2.0-15.0]
**Treatment – n (%)**	
**Oxygen therapy**	2 (6.9)
**Lopinavir/Ritonavir**	4 (13.8)
**Hydroxychloroquine**	4 (13.8)
**Corticosteroids**	1 (3.4)
**Heparin**	3 (10.3)
**Antibiotics**	7 (24.1)
Lymphocytes (cells/mm^3^)*	1400 [600-2600]
Hemoglobin (g/dL)*	11.8 [9.7-14.0]

Values are taken at baseline. Continuous variables are expressed as the medians and interquartile ranges [IQR]. Categorical variables are expressed as numbers and percentages. Percentages have been rounded per convention. *Data were only available from 22 SARS-CoV2 infected mothers. COVID-19, coronavirus disease 2019; SARS-CoV2, severe acute respiratory syndrome coronavirus 2; PCR, polymerase chain reaction.

**Table 2:** Exposed SARS-CoV2 newborns’ characteristics.

Parameters	Values
**Exposed newborn - n**	25
**Gestational age (weeks)**	39.4 [29.5-41.2]
**Mode of delivery n (%)**	
**Eutocic**	11 (44)
**Caesarean**	10 (40)
**Instrumental**	4 (16)
**Gender (male) n (%)**	13 (52)
**Breastfeeding n (%)**	16 (66.7)
**APGAR1**	9.0 [5.0-10.0]
**APGAR5**	10.0 [8.0-10.0]
**Weight (Kg)**	3.2 [1.1-3.9]
**Height (cm)**	50.0 [46.0-54.0]
**Head circumference (cm)**	34.0 [29.0-36.0]
**Symptoms - n (%)**	8 (33.3)
**Several symptoms**	4 (25)
**Mild symptoms**	4 (25)
**Time of symptoms (days)**	10.0 [0.0-50.0]
**Age onset symptoms (days)**	0.0 [0.0-5.0]
**Admitted to NICU**	5 (20)
**Time in NICU (days)**	9.0 [1.0-85.0]
**Treatment - n (%)**	2 (8.3)
**Time of treatment (days)**	4.0 [3.0-5.0]
**Oxygen therapy**	4 (16)
**Surfactant**	3 (12)
**SARS-CoV2 Serology (+) n (%)**	
**Postnatal IgG (+)**	3 (12)

Values are taken at baseline after childbirth. Continuous variables are expressed as the medians and interquartile ranges [IQR]. Categorical variables are expressed as numbers and percentages. Percentages have been rounded per convention. SARS-CoV2, severe acute respiratory syndrome coronavirus 2; PCR, polymerase chain reaction; APGAR, Appearance, Pulse, Grimace, Activity, and Respiration; APGAR1, 1-minute APGAR; APGAR5, 5-minute APGAR.; NICU, Neonatal Intensive Care Unit.

### Different Cytokine Levels in SARS-CoV2 Mothers

**Figure 1:**
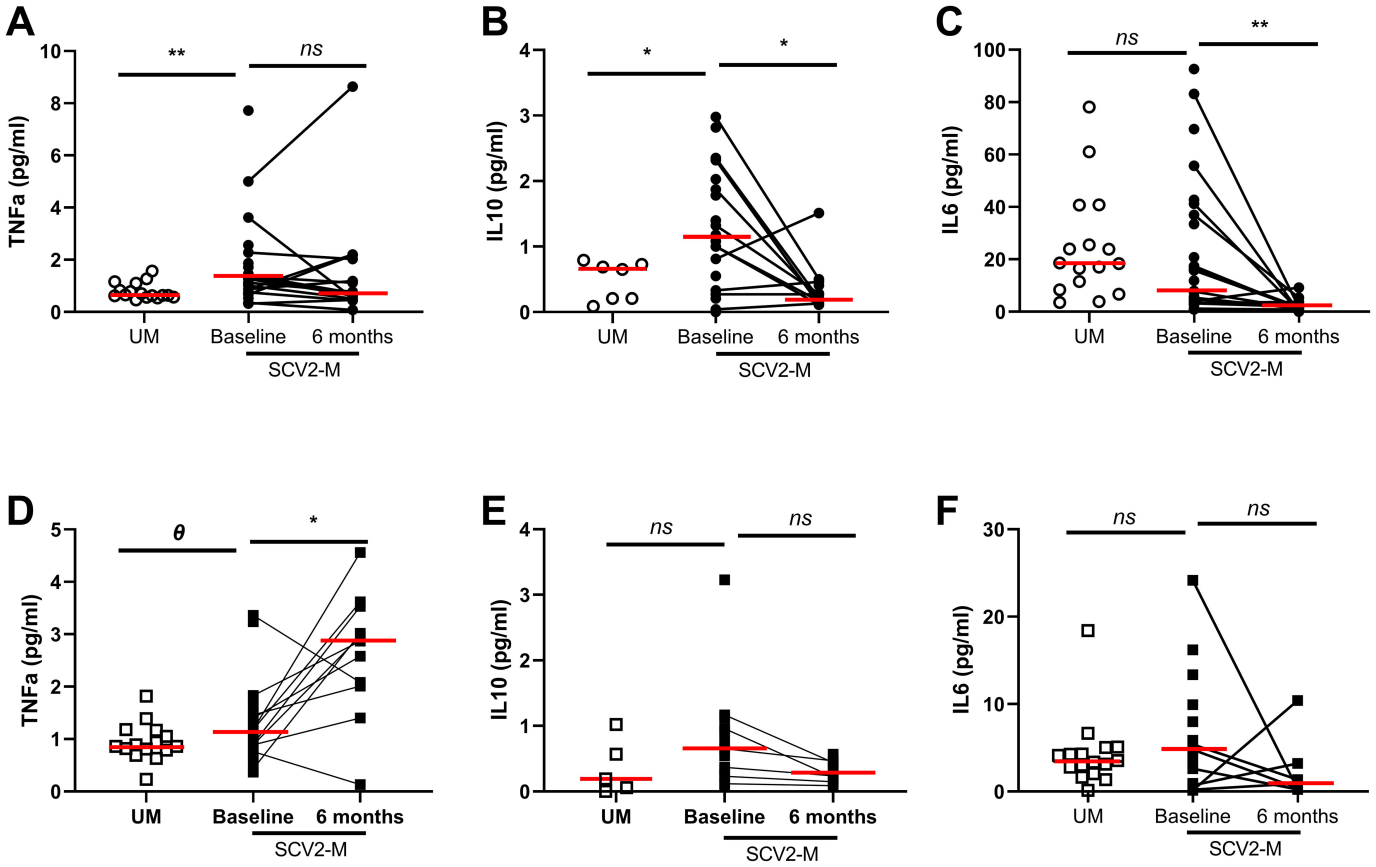
Soluble pro/anti-inflammatory cytokine levels in plasma. Differences at baseline and 6 months later. Soluble TNF-α, IL-10 and IL-6 levels from mothers and newborns’ plasma at baseline and after 6 months (A-F); Mann-Whitney U-test was used to compare groups. Wilcoxon test was conducted to compare paired events. SCV2-M, SARS-CoV2 mothers’ group; UM, Uninfected mothers’ group. **p ≤ 0.01, *p<0.05, *Ɵ 0.05≤p ≤ 0.1*, *ns p>0.1*.

Soluble TNFα (*p*=0.004) and IL-10 (*p*=0.03) levels were increased at baseline in SARS-CoV2 infected mothers compared to uninfected mothers. No significant differences were observed comparing soluble IL-6 levels in mothers, but the levels of this cytokine correlated directly with age (r=0.33; p=0.04) (*data not shown*). In the case of soluble IL-10 and IL-6, after six months the levels significantly decreased in SARS-CoV2 infected mothers (*p*=0.03 and *p*=0.002 respectively) (**Figures 1A-C**). Regarding newborns, such as SARS-CoV2 infected mothers, those exposed to the virus had higher soluble TNFα (*p*=0.07) and IL-10 levels (*p*=0.16) in cord plasma at baseline than non-exposed newborns (**Figures 1D, E**). However, soluble TNFα levels (*p*=0.03) significantly increased after six months. No significant differences were found for soluble IL-6 levels either at baseline or at the follow-up (*p*=0.60 and *p*=0.57, respectively) on exposed newborns (**Figure 1F**). The levels of IL-17 were below the limit of detection and excluded from further analysis.

### Different NK and Monocytes Cell Subsets Distribution and Activation, Maturation and Endothelial Adhesion Markers in SARS-CoV2 Infected Mothers and Exposed Newborns

**Figure 2:**
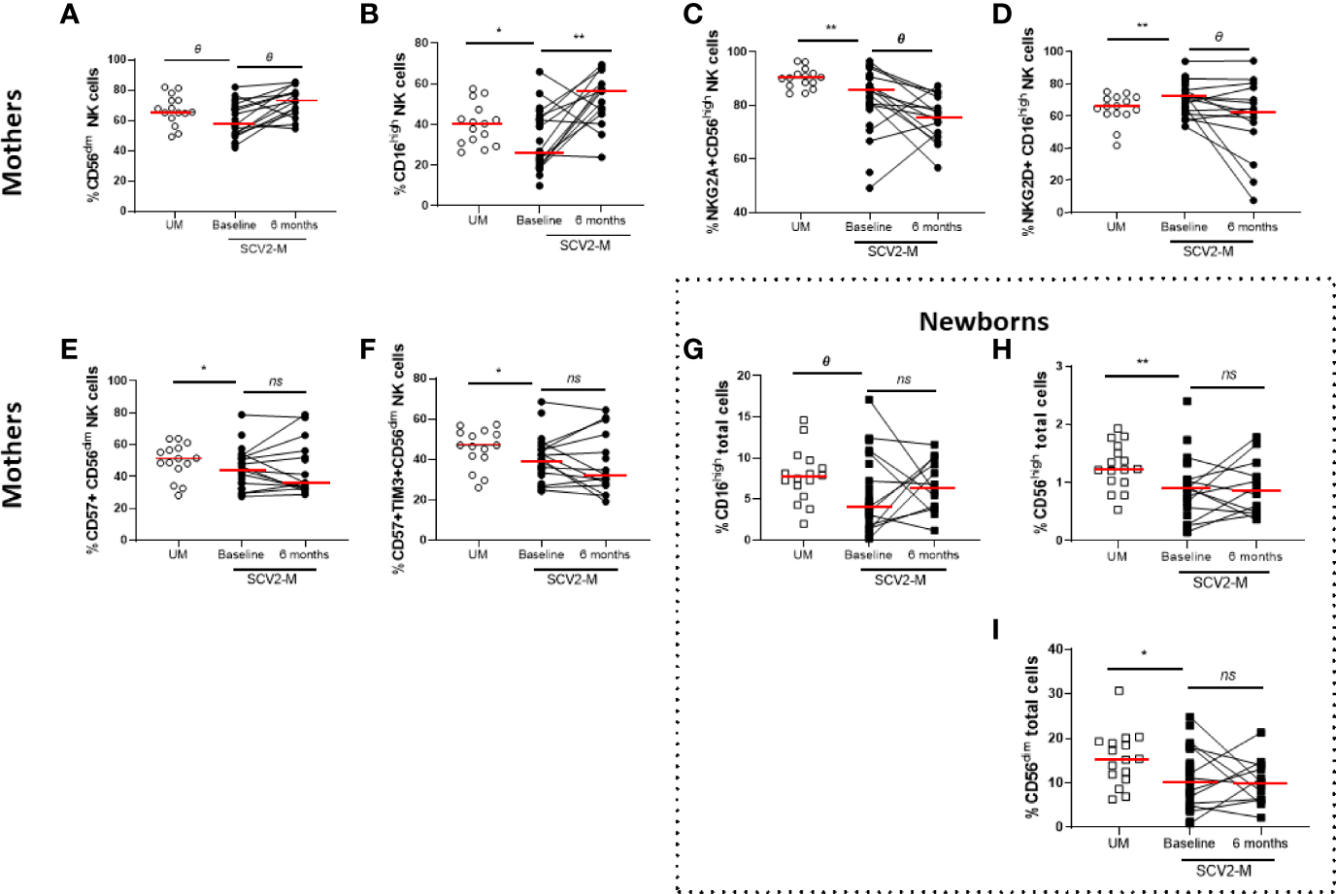
Frequency of NK cell subsets, activation and inhibition receptors and maturation markers. Differences at baseline and 6 months later. Frequency of CD56^dim^ and CD16^high^ NK cell subsets in mothers **(A, B)**. NKG2A, NKG2D expression in CD56^high^ and CD16^high^ NK cell subsets, respectively **(C, D)**; CD57 and CD57, TIM3 co-expression in CD56^dim^ NK cell subsets **(E, F)**. Frequency of CD16^high^, CD56^high^ and CD56^dim^ total cells in newborns **(G–I)**. Mann-Whitney U-test was used to compare groups. Wilcoxon test was conducted to compare paired events. SCV2-M, SARS-CoV2 mothers’ group; UM, Uninfected mothers’ group. **p ≤ 0.01, *p<0.05, Ɵ 0.05≤p ≤ 0.1, ns p>0.1.

Regarding NK cell subsets, SARS-CoV-2 infected mothers had significantly lower levels of CD56^dim^ (*p*=0.05) and CD16^high^ *(p*=0.04) NK cell subsets at baseline than those of uninfected mothers. In the case of CD16^high^ NK cell subset, after six months the levels significantly increased (*p*=0.004) in SARS-CoV2 infected mothers (**Figures 2A, B**). Also, SARS-CoV-2 infected mothers showed low levels of the inhibiting receptor NKG2A in CD56^high^ (*p*=0.01) NK cell subset and high expression of the cytolytic C-type lectin-like activating receptor NKG2D in CD16^high^ (*p*=0.01) NK cell subset at baseline compared to uninfected mothers. No changes were observed after 6 months (*p*=0.05 and *p*=0.09, respectively) (**Figures 2C, D**). Similarly, the decreased levels of the maturation markers CD57 (*p*=0.04) and CD57/TIM3 co-expression (*p*=0.02) found on CD56^dim^ NK cell subset at baseline from SARS-CoV2 infected mothers showed no differences 6 months later (**Figures 2E, F**). The total NK cells and different subset distribution was analyzed relative to the total live PBMCs in cord cells from newborns (**Supplementary Table 2**) and interestingly, we observed that CD16^high^ (*p*=0.05), CD56^high^ (*p*=0.007) and CD56^dim^ (*p*=0.04) total NK cells were decreased at baseline in cord cells in exposed newborns compared to non-exposed, and no significant changes were observed 6 months later (**Figures 2G–I**).

Interestingly, soluble IL-10 levels from all included mothers strongly and inversely correlated with the activation marker CD158b in CD16^high^ NK cell subset at baseline and a direct significant correlation was also obtained between soluble TNFα levels and TIM-3 Medium Fluorescence Intensity (MFI) in CD56^dim^ NK cell subset (**Supplementary Figures 2A, B**).

According to the expression of CD14 and CD16, monocyte cells were classified in three subsets: patrolling (CD16^high^ CD14^dim^), intermediate (CD16^dim^ CD14^high^) and classical (CD16^neg^ CD14^high^). SARS-CoV2 infected mothers showed increased levels of CD62L expression in the classical subset (*p*=0.03), elevated intermediate monocytes proportion (*p*=0.03) and decreased CD40 and CD49d expression in patrolling and classical subsets (*p*=0.03 and *p*=0.03, respectively) (**Supplementary Figures 2C–F**). Data concerning monocytes was not available after 6 months. Regarding newborns, the frequency of the three monocyte subsets was quantified relative to the total live PBMCs in cord cells at baseline, and we observed that exposed children presented lower frequency of total and subset monocytes than the unexposed. Total classical monocytes showed a significant decreased at follow-up-time-point, 6 months later (*p*=0.02) (**Supplementary Figures 2G–J** and **Supplementary Table 2**).

### SARS-CoV2 Infected Mothers Show Increased Activation and Exhaustion Markers in T Lymphocytes

**Figure 3:**
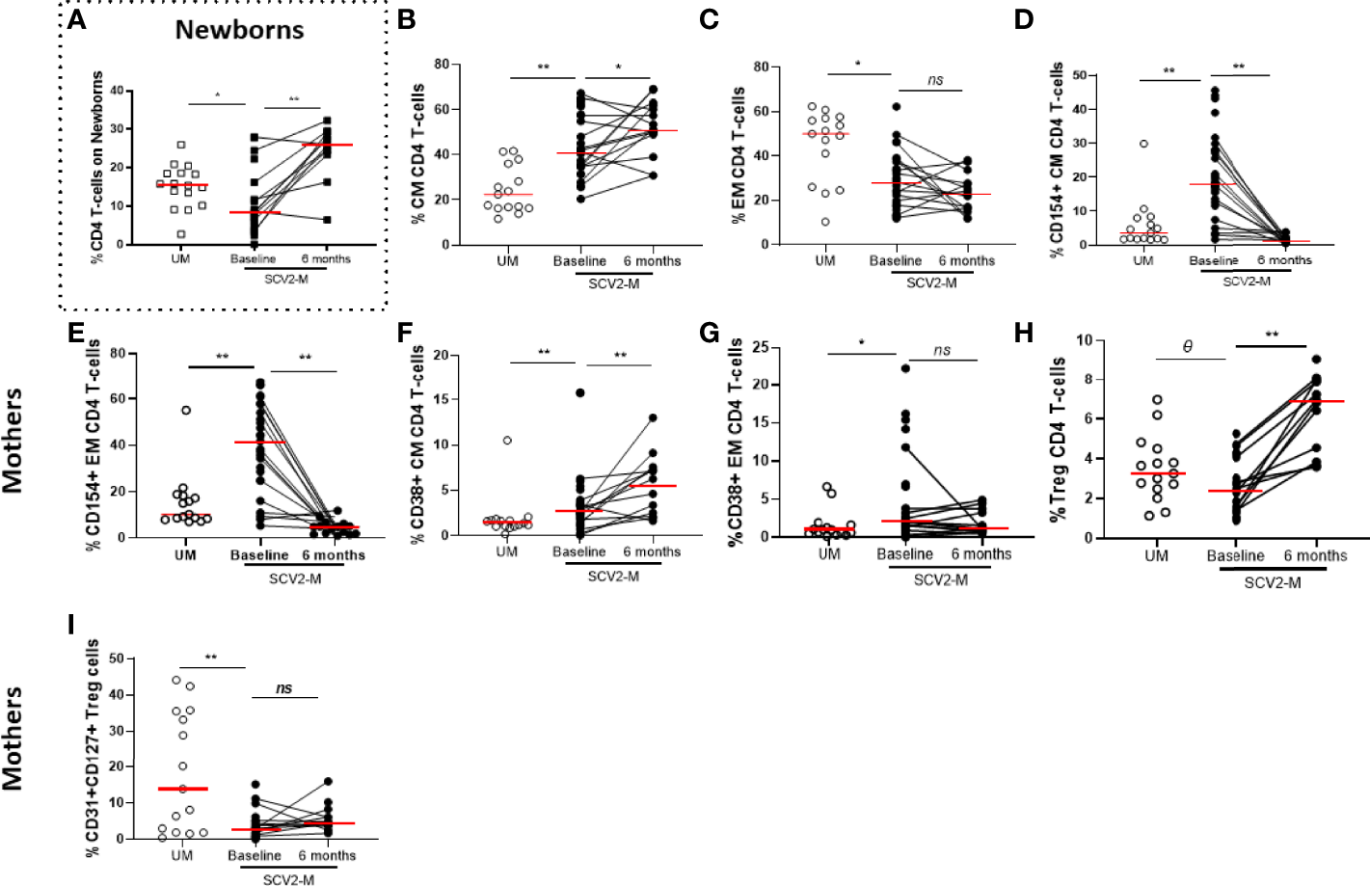
Maturation profile and activation markers on CD4 T lymphocytes and frequency of Treg cells. Frequency of total CD4 T lymphocytes in newborns **(A)**. Differences in Central and Effector Memory (CM and EM, respectively) subsets distribution in CD4 T cells **(B, C)**; activation markers in CM and EM CD4 T-cell memory subsets **(D–G)**; Treg cells proportion **(H)** and CD31, CD127 co-expression in Treg cells **(I)**. Mann-Whitney U-test was used to compare groups. Wilcoxon test was conducted to compare paired events. SCV2-M, SARS-CoV2 mothers’ group; UM, Uninfected mothers’ group. **p ≤ 0.01, *p<0.05, *Ɵ 0.05≤p ≤ 0.1*, *ns p>0.1*.

The T lymphocyte immunophenotyping from exposed newborns’ cord cells included markers for determining the frequency of CD4 and CD8 total T-cells relative to the total live PBMCs and naïve and recent thymic emigrants’ subsets (CD45RA and CD31 respectively) (**Supplementary Table 2**) and only the total frequency of CD4 T-cells (*p*=0.02) was decreased in SARS-CoV2 exposed newborns compared to non-exposed, but those levels significantly increased (*p*=0.002) 6 months later (**Figure 3A**). In mothers, at baseline, SARS-CoV2 infected mothers had higher frequency of Central Memory (CM, CD45RA+CD27-) and lower Effector Memory (EM, CD45RA-CD27-) CD4 T-cell subsets (*p*<0.001 and *p*=0.017 respectively) compared to uninfected mothers. The frequency of CM increased (*p*=0.035) after 6 months, while no changes were observed in EM subset distribution (**Figures 3B, C**). No significant differences (in all cases, p≥0.05) were observed in naïve (CD45RA+CD27+) and Terminally Differentiated (TemRA, CD45RA+CD27-) CD4 T-cell memory subsets or in the distribution of CD8 T-cell memory subsets. Regarding activation markers, we observed, at baseline, higher expression of CD154 and CD38 in total CD4 T-cells (*p*=0.018 and *p*<0.001, respectively). Also, we found in CM and EM CD4 T-cell subsets, increased levels of CD154 (*p*<0.001 and *p*<0.001 respectively) and CD38 expression (*p*=0.006 and *p*=0.017 respectively) from SARS-CoV2 infected mothers compared with uninfected mothers (**Figures 3D–G**). However, this activation decreased over the time only in the case of CD154 expression in CM and EM CD4 T-cell subsets (*p*<0.001 for both comparisons). Interestingly, the levels of CD154 and CD137 expression in CD4 T-cell subsets directly correlated with soluble TNF-α levels in all women studied and in the case of the activation marker HLA-DR expressed in CM CD8 T-cells, the levels of all women correlated inversely with soluble IL-10 (**Supplementary figures 3 A–F**). No significant differences (*p*≥0.05) were observed in the co-expression of the activation markers HLA-DR and CD38 in all T-cell subsets *(data not shown*). SARS-CoV2 infected mothers showed decreased frequency of Treg cells (defined as CD4 T-cells positive for CD25 and Fox3) (*p*=0.085) compared to uninfected mothers at baseline, but the levels significantly restored (*p*=0.001) 6 months later (**Figure 3H**). Interestingly, the frequency of Treg expressing the recent thymic emigrant marker CD31 and the IL-7 receptor (CD127) was lower (*p*=0.001) on SARS-CoV2 infected mothers compared to uninfected mothers and the levels remain decreased 6 months later (**Figure 3I**).

**Figure 4:**
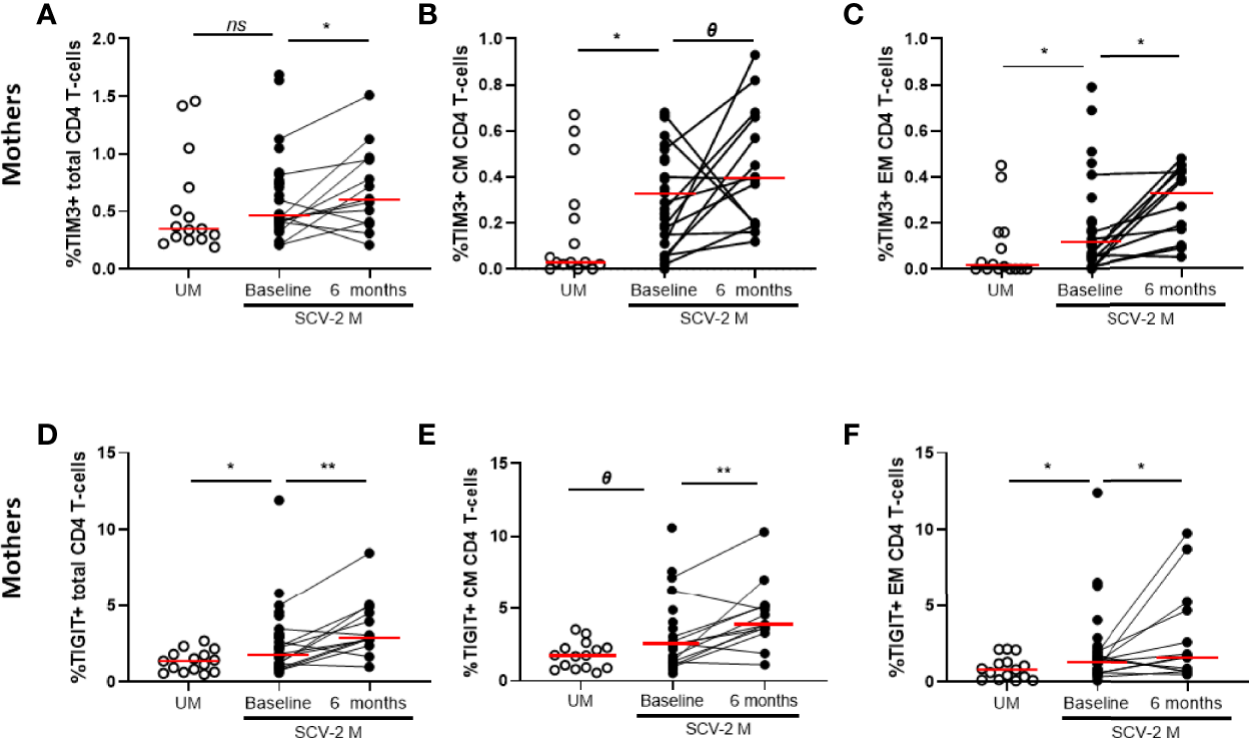
Exhaustion markers on CD4 T-cells subsets. TIM-3 expression in total CD4 T cells **(A)** and in Central and Effector Memory (CM and EM, respectively) **(B, C)** and TIGIT expression in total **(D)** and CM and EM CD4 T cells **(E, F)**. Mann-Whitney U-test was used to compare groups. Wilcoxon test was conducted to compare paired events. SCV2-M, SARS-CoV2 mothers’ group; UM, Uninfected mothers’ group. **p ≤ 0.01, *p<0.05, *Ɵ 0.05≤p ≤ 0.1*, *ns p>0.1*.

The T-cell characterization also included the analysis of CD57 for senescence and PD1, TIM-3, TIGIT and LAG-3 for cell exhaustion. At baseline, high levels of TIM-3 and TIGIT in total (*p*=0.132 and *p*=0.026, respectively), CM (*p*=0.011 and *p*=0.05, respectively) and EM (*p*=0.021 and *p*=0.029, respectively) CD4 T-cell subsets were found in SARS-CoV2 infected mothers compared to uninfected mothers, in all cases those exhaustion marker levels increased also 6 months later (**Figures 4A–F**). Similar results were obtained for TIM-3 and TIGIT expression on total, CM and EM CD8 T-cells and the levels remain also elevated after 6 months (*data not shown*).

The multiple exhausted phenotypes defined by the simultaneous expression of more than one of the examined exhaustion markers was also studied. SARS-CoV2 infected mothers exhibited increased levels only of CD4 T-cells expressing the four analysed exhaustion markers (TIM3+PD1+TIGIT+LAG3+) in total and CM subset (*p*=0.022 and *p*=0.016, respectively) compared to uninfected mothers. Comparing baseline to 6 months of the follow-up, the levels significantly increased (*p*=0.002 and *p*=0.004, respectively) in SARS-CoV2 infected mothers (**Supplementary Figure 4A**). This trend was also observed at baseline, when specific combinations of three exhaustion markers was examined in CM CD8 T-cells (*p*=0.019) (**Supplementary Figure 4B**). In the case of CD8 T-cell subsets, while there was no significant combination of three markers, the combination that included the co-expression of TIGIT and TIM3 (TIM3+PD1-TIGIT+LAG3-) was increased in all CD8 T-cell subsets (total, *p*=0.024; CM, *p*=0.001; EM, *p*=0.001 and TemRA, *p*=0.006) on SARS-CoV2 infected mothers compared to uninfected mothers at baseline (**Supplementary Figures 4C, D**). Interestingly, the peculiar exhaustion phenotype (LAG3-PD1+TIGIT-TIM3-) from total CD8 T-cells inversely correlated with the soluble IL-6 levels at baseline on all mothers (**Supplementary Figure 4E**).

## Discussion

This is the first longitudinal study on the changes in the immune defenses induced by SARS-CoV-2 infection in pregnant women and their newborns. Due to the unique immune state at pregnancy, we compared the cellular and humoral defenses of the SARS-CoV-2 infected mothers and their newborns with those of uninfected mothers and their newborns.

No SARS-CoV2 vertical transmission was observed in our cohort accordingly to most of screenings carried out in pregnant women and neonates (18, 19, 31). Nonetheless, our study confirmed previous investigations in which SARS-CoV-2 infection leads to increased soluble pro- and anti-inflammatory cytokines, called “cytokine storm” (32–34). We observed, at the moment of childbirth, high soluble TNF-α and IL-10 levels in SARS-CoV2 infected mothers while soluble IL-6 levels were significantly decreased in those mothers, contrary to what we expected (35–37). Nevertheless, other studies have suggested that IL-10 may control the intensity of the inflammatory response in which IL-6 participates (38). Both cytokines could be implied in maintaining a balance and preventing the impaired and exacerbated inflammatory response (39). After SARS-Cov2 infection, we observed a reduction in the inflammatory response defined by IL-6 and TNF-α and anti-inflammatory soluble IL-10 cytokine levels were decreased six months later too. A similar tendency of dysregulated cytokines was found in plasma cord samples from newborns, reflecting the possible influence of their mothers’ inflammatory status during COVID-19 infection, which in previous publications has been related to placental immune activation (40) although the transfer of proinflammatory cytokines across term placenta remains still unclear (27, 41). However, as occurs with HIV-1-exposed uninfected newborns, the altered levels of soluble cytokines, such as TNF-α and IL-10 at birth, remains dysregulated after six months (26).

Regarding innate immunity, a dysregulated monocyte response has been reported to be involved in the pathogenesis and cytokine storm during COVID-19 infection (42). Our results support those observations, we observed altered monocyte subsets expressing activation markers in SARS-CoV2 infected mothers. Additionally, decreased proportions of CD56^dim^ and CD16^high^ NK cell subsets were found at baseline in SARS-CoV2 infected mothers. This agrees with previous observations carried out in general infected population (29), in which the declined in NK cell is compensated by an increased cytotoxic potential. Interestingly, NK cells from our SARS-CoV2 infected mothers also expressed high levels of the activating receptor NKG2D involved in inducing a cytotoxic activity. The decreased proportion of NK cells in pregnant women may reflect a possible redistribution of these cells to infected anatomical places such as bronchoalveolar tissue, where they may migrate to eliminate virus in SARS-CoV2 infected mothers (43–45). Interestingly, only part of the innate immune cell subsets in the peripheral blood of SARS-CoV2 infected pregnant women recovering from COVID-19 reversed to normal levels at six months after childbirth accordingly previous observations on pregnant women (28).

CD4 and CD8 T lymphocyte subsets play an important role in SARS-CoV2 immune response and a marked lymphopenia characterized severe SARS-CoV2 infection (46). In our study, we did not find any evidence of lymphopenia in infected pregnant women compared to non-infected pregnant women probably due to the low number of severe cases included. However, we observed an increase of Central Memory CD4 subset and decrease of Effector Memory CD4 T-cells in SARS-CoV2 mothers compared to uninfected mothers that unchanged 6 months later according to previous results (47, 48). In the case of increased Central Memory cells, it may indicate appearance of immune memory in patients with COVID-19 infection. Regarding the Effector Memory subset, the reduction in the percentage of these cells could be explained by their recruitment into infected organs, such as the lungs, or by cell damage caused by the massive release of inflammatory mediators in response to infection (33, 49, 50).

T-cells of SARS-CoV2 infected mothers from our study were also characterized by an increased activation and exhaustion profile. High proportion of activation and exhaustion makers on T cell in COVID-19 patients have been associated with an unfavorable disease outcome (51). These data indicate that SARS-CoV2, similarly to some chronic infections, damages the function of T cells and promotes an exacerbated activation and exhaustion on these cells that could diminish host antiviral immunity too. Further studies with a longer follow-up will determine the clinical consequences of the persistent exhaustion levels we also found six months after childbirth, which could lead to what is known as “long COVID syndrome” (52).

This study identifies changes suggestive of a fetal immune response after SARS-CoV2 maternal infection in the absence of vertical viral transmission and suggests potential trans-placental immune implications of maternal infection beyond vertical transmission. We identified a transient response to maternal inflammation in cord plasma and immune cell functionality in SARS-CoV2 exposed newborns, indicating some immune imprinting at childbirth (30), that after six months seems to restore during newborns’ immune system development. This is the case of the lymphopenia on CD4 T cells we observed in SARS-CoV2 exposed newborns in cord blood that significantly increased after six months. The high prevalence of preterm birth, prematurity and fetal growth restriction (53–55) that characterized SARS-CoV2 infected women could explain the altered proportions of those cells among others. In the case of NK cell subsets from SARS-CoV2 exposed newborns, we observed low levels in blood cord samples and six months later confirming a certain impact on newborns due to the inflammatory response against the virus. Further a long-term follow-up of SARS-CoV2 exposed newborns in our study would elucidate if maternal inflammation during COVID-19 infection has a long-lasting impact on the child development.

Our study was observational and had some limitations. The main limitation is that the healthy control, uninfected mothers, and unexposed newborns’ groups could not be set at follow-up time point, 6 months later, due to the epidemiological situation of SARS-CoV-2 pandemic during the study. Some of those uninfected mothers recruited at childbirth suffered from the infection during that time, received one vaccine dose or were fully vaccinated. The sample size was relatively small and hospitalized/severe pregnant patients accounted for only a small fraction of the participants and this explain that no detailed biochemical or blood count analysis were assessed. Due to that, we did not have information on absolute cell numbers. More severe maternal infections could result in more dramatic or different newborns’ immune signature. Additionally, pregnant women enrolled in our study developed symptoms at different gestational age. The time from infection to delivery and cord blood collection may also affect the immune phenotypes as we described on mothers’ and newborns’ immune phenotypes. Our research provides valuable data for supporting the vaccination in the poorly studied population of pregnant women. Further approaches that include a group of vaccinated women infected by SARS-CoV2 during pregnancy will elucidate the importance of expanding the vaccine administration to this risk group.

## Conclusions

SARS-CoV2 infection during pregnancy shows differences in immunological components that could lead newborns to future clinical implications after birth. However, SARS-CoV2 exposed 6-months-old newborns showed no immune misbalance, whereas the infected mothers maintain increased activation and exhaustion levels in T-cell after 6 months.

## Data Availability Statement

The raw data supporting the conclusions on this article will be made available by the authors, without undue reservation.

## Ethics Statement

The studies involving human participants were reviewed and approved by Ethics Committee of HGUGM (Ref: IRB 0000605). Written informed consent was obtained from all the mothers and newborns’ legal guardians before inclusion in the cohort GESNEO-COVID.

## Author Contributions

EV-A: designed the study, performed the research, analysed the data, designed the figures, and wrote the paper. LT-D: designed the study, performed the research, analysed the data, designed the figures, and wrote the paper. IC: designed the study, recruited and characterized the patients, collected samples and data, and reviewed the manuscript. SV-V: recruited and characterized the patients, collected samples and data, and reviewed the manuscript. MM-C: recruited and characterized the patients. ER-L: recruited and characterized the patients. JS-L: recruited and characterized the patients. MS-S: recruited and characterized the patients. DA-A: recruited and characterized the patients. AH-L: recruited and characterized the patients. BS-G: recruited and characterized the patients. JAL-L: recruited and characterized the patients. PM: contributed with laboratory’s determination. MS-L: recruited and characterized the patients. MLN: designed the study, recruited and characterized the patients, collected samples and data, interpreted the results, wrote and reviewed the manuscript, and gave the final approval of the manuscript. MAM-F: designed the study, performed the research, analysed the data, interpreted the results, wrote and reviewed the manuscript, and gave the final approval of the manuscript. All authors have critically reviewed and approved the final manuscript.

## Funding

This work has been (partially) supported by the Instituto de Salud Carlos III (ISCII; grant numbers COV20_00808), the RD16/0025/0019 project as part of Acción Estratégica en Salud, Plan Nacional de Investigación Científica, Desarrollo e Innovación Tecnológica (2020-2022) and co-financed by Instituto de Salud Carlos III (Subdirección General de Evaluación) and Fondo Europeo de Desarrollo Regional (FEDER), RETIC PT17/0015/0042, Fondo de Investigación Sanitaria (FIS) 2020-2022 (grant number PI19/01638) for MAMF. Moreover, this work has been supported partially by a EUROPARTNER: Strengthening and spreading international partnership activities of the Faculty of Biology and Environmental Protection for interdisciplinary research and innovation of the University of Lodz Programme: NAWA International Academic Partnership Programme (MAMF). This article/publication is based upon work from COST Action CA 17140 “Cancer Nanomedicine from the Bench to the Bedside” supported by COST (European Cooperation in Science and Technology) 2018-2022 (MAMF). EV-A was supported by the Instituto de Salud Carlos III and the Fondo Europeo de Desarrollo Regional (grant number PI19/01638). LT-D was supported by the Instituto de Salud Carlos III (ISCIII) under grant agreement “CD20/00025” through the Sara Borrell Program and by GeSIDA through the “Premio para Jóvenes Investigadores 2021″. HL-A is funded by the Spanish Ministry of Science and Innovation-Instituto de Salud Carlos III (ISCIII) and Fondos FEDER through the Río Hortega Program (CM20/00128).

## Conflict of Interest Statement

The authors declare that the research was conducted in the absence of any commercial or financial relationships that could be construed as a potential conflict of interest.

## Publisher’s Note

All claims expressed in this article are solely those of the authors and do not necessarily represent those of their affiliated organizations, or those of the publisher, the editors and the reviewers. Any product that may be evaluated in this article, or claim that may be made by its manufacturer, is not guaranteed or endorsed by the publisher.

## Supplementary Material

Supplementary Figure 1 Schematic diagram of lymphocytes, NK cells and monocytes gating strategy of an uninfected mother. Lymphocyte gate strategy (A). NK and subsets gate strategy (B). Monocyte and subsets gate strategy (C). Isotype controls (in red) have been used to analyze the expression of markers (blue). Representative plots are shown in each cell type. Viab: viability.

Supplementary Figure 2 NK cells correlations with cytokine levels at baseline and phenotype of monocytes subsets. Correlation of CD158b in CD16^high^ NK cells and soluble IL-10 **(A)**; correlation of median-intensity fluorescence (MFI) TIM-3 in CD56^dim^ NK cells and soluble TNF-α **(B)**. CD62L expression in classical monocytes (CD16^neg^ CD14^high^), intermediate monocytes proportion (CD16^dim^ CD14^high^) and CD40 expression in patrolling monocytes (CD16^high^ CD14^dim^) **(C-E)**; CD49 expression in classical monocytes **(F)**; frequency of total monocytes (CD14+HLA-DR+) and classical, intermediate and patrolling monocytes cell subsets in newborns **(G-J)**. Mann-Whitney U-test was used to compare groups. Wilcoxon test was conducted to compare paired events. The Spearman ρ correlation coefficient test was used. SCV2-M are highlighted with black dots and white dots represent UM. SCV2-M, SARS-CoV2 mothers’ group; UM, Uninfected mothers’ group. ******** *p ≤ 0.01*, ******* *p<0.05*, ***Ɵ*** *0.05≤p ≤ 0.1*, ***ns*** *p>0.1*

Supplementary Figure 3 Correlations between soluble cytokine levels and activation markers on T-cells from all mothers at baseline. Correlation of CD154 and CD137 markers in total, Central and Effector Memory (CM and EM, respectively) CD4 T-cell subsets with soluble TNF-α **(A-E)**. Correlations of HLA-DR markers in CM CD8 T-cell subset with soluble IL-10 levels **(F)**. IL-10 data were only available from 24 mothers. SCV2-M are highlighted with black dots and white dots represent UM. Values are taken at baseline. The Spearman ρ correlation coefficient test was used.

Supplementary Figure 4 Multiple exhaustion phenotype on T-cells at baseline and 6 months later and association between exhaustion phenotype on CD8 T-cells and soluble cytokine levels at baseline. Simultaneous expression of four exhaustion markers (TIM3+PD1+TIGIT+LAG3+) in total and Central Memory (CM) CD4 T-cell subsets **(A)**. Combination of three exhaustion markers (TIM3+PD1+TIGIT+LAG3-) in CM CD8 T-cells **(B)**. Expression of three exhaustion markers combination (TIM3+PD1-TIGIT+LAG3-) in total CD8-T cells and Central, Effector and Terminally Differentiated Memory subsets (CM, EM and TemRA) **(C, D)**. Correlation between exhaustion markers (LAG3-PD1+TIGIT-TIM3-) in total CD8 T-cells and soluble IL-6 **(E)**. Mann-Whitney U-test was used to compare groups. Wilcoxon test was conducted to compare paired events. The Spearman ρ correlation coefficient test was used. SCV2-M are highlighted with black dots and white dots represent UM. SCV2-M, SARS-CoV2 mothers’ group; UM, Uninfected mothers’ group. ***p ≤ 0.01, *p<0.05, Ɵ 0.05≤p ≤ 0.1, ns p>0.1*
