# Corrigendum: SARS-CoV2 infection during pregnancy causes persistent immune abnormalities in women without affecting the newborns

**DOI:** 10.3389/fimmu.2025.1574788

**Published:** 2025-02-27

**Authors:** Elena Vazquez-Alejo, Laura Tarancon-Diez, Itzíar Carrasco, Sara Vigil-Vázquez, Mar Muñoz-Chapuli, Elena Rincón-López, Jesús Saavedra-Lozano, Mar Santos-Sebastián, David Aguilera-Alonso, Alicia Hernanz-Lobo, Begoña Santiago-García, Juan Antonio de León-Luis, Patricia Muñoz, Manuel Sánchez-Luna, María Luisa Navarro, Mª Ángeles Muñoz-Fernández

**Affiliations:** ^1^ Immunology Section, Laboratory of ImmunoBiology Molecular, Hospital General Universitario Gregorio Marañón (HGUGM), HIV-HGM BioBank, Madrid, Spain; ^2^ Infectious Diseases in Paediatric Population, Gregorio Marañón Research Institute (IiSGM) and University Hospital, Madrid, Spain; ^3^ Infectious Diseases Section, Department of Paediatrics, Hospital General Universitario Gregorio Marañón (HGUGM), Madrid, Spain; ^4^ Department of Obstetrics and Gynecology, Hospital General Universitario Gregorio Marañón (HGUGM), Madrid, Spain; ^5^ Department of Neonatology, Hospital General Universitario Gregorio Marañón (HGUGM), Madrid, Spain; ^6^ Department of Clinical Microbiology and Infectious Diseases, Hospital General Universitario Gregorio Marañón (HGUGM), CIBER Enfermedades Respiratorias (CIBERES), Madrid, Spain; ^7^ Faculty of Medicine, Universidad Complutense de Madrid, Madrid, Spain; ^8^ CIBER of Infectious Diseases (CIBERINFEC), Madrid, Spain

**Keywords:** SARS-CoV2, pregnancy, SARS-CoV2 exposed newborns, immune system, longitudinal analysis

In the published article, there was an error in **Figure 1** as published. The graphic 1E of **Figure 1** (IL-10 cytokine levels in newborns) visually corresponds to the analysis of the graphic 1B (IL-10 cytokine levels) in mothers. Although the statistical analysis is correctly indicated, the data representation for that cytokine in newborns is incorrect. While this error does not affect any of the results presented in the article, as the data analysis and discussion were based on the correct statistical results, it is nonetheless a visual error. The corrected **Figure 1** and its caption appear below.

**Figure 1:**
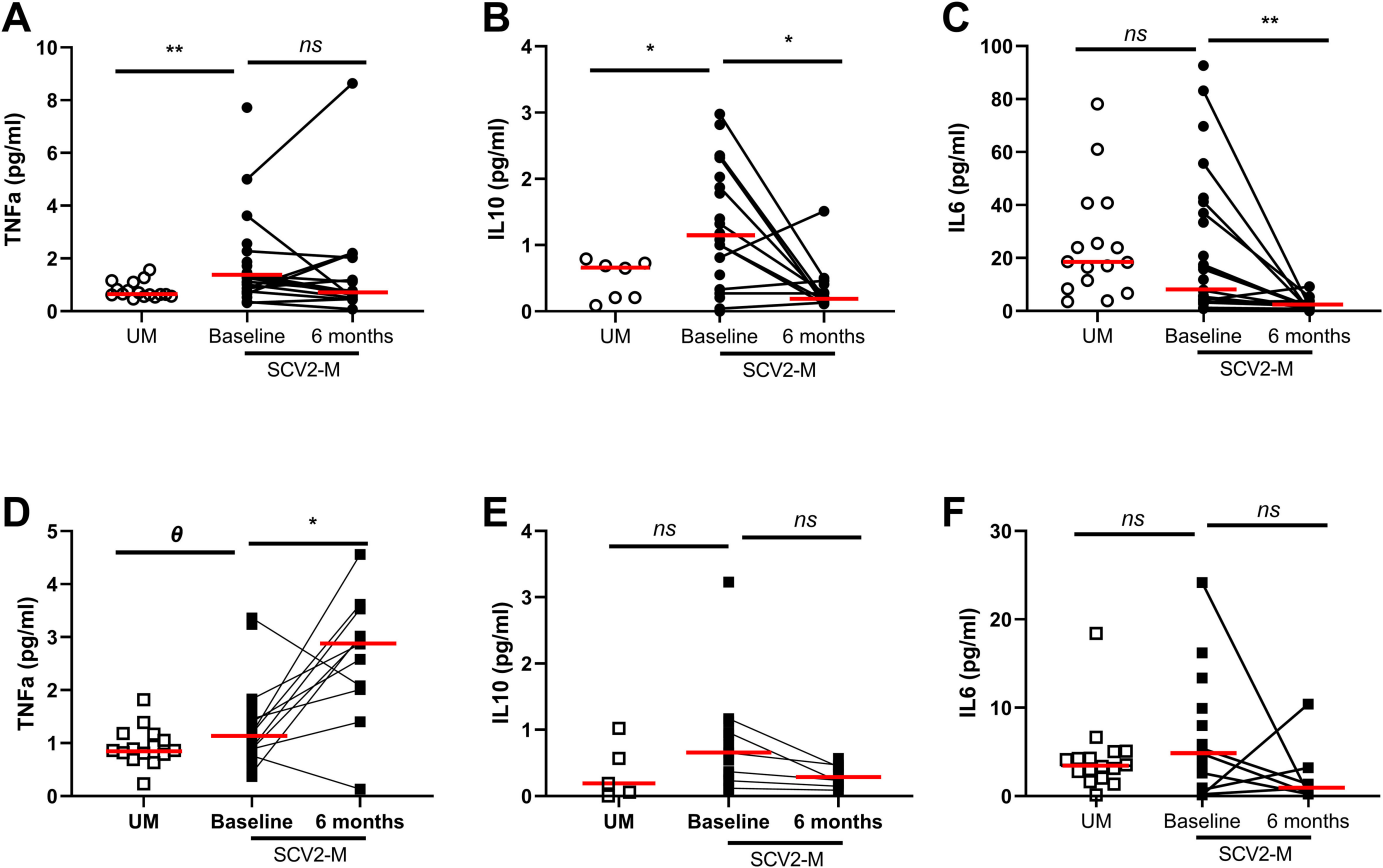
Soluble pro/anti-inflammatory cytokine levels in plasma. Differences at baseline and 6 months later. Soluble TNF-α, IL-10 and IL-6 levels from mothers and newborns’ plasma at baseline and after 6 months **(A–F)**; Mann-Whitney U-test was used to compare groups. Wilcoxon test was conducted to compare paired events. SCV2-M, SARS-CoV2 mothers’ group; UM, Uninfected mothers’ group. **p ≤ 0.01, *p<0.05, *Ɵ* 0.05≤*p* ≤ 0.1, ns p>0.1.

The authors apologize for this error and state that this does not change the scientific conclusions of the article in any way. The original article has been updated.

