# Supplementary figures and images for: SARS-CoV2 Infection During Pregnancy Causes Persistent Immune Abnormalities in Women Without Affecting the Newborns
Source: Front Immunol. 2022 Jul 14;13:947549. doi: 10.3389/fimmu.2022.947549 (PMC9330630; doi:10.3389/fimmu.2022.947549)

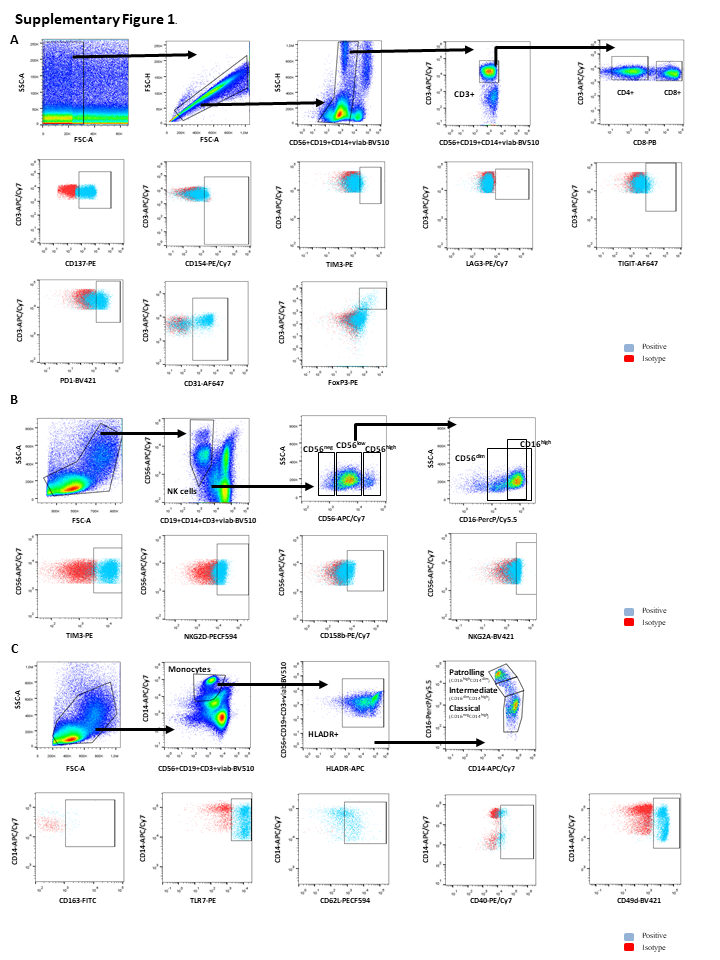

Supplement: Supplementary Figure 1 — Schematic diagram of lymphocytes, NK cells and monocytes gating strategy of an uninfected mother. Lymphocyte gate strategy (A). NK and subsets gate strategy (B). Monocyte and subsets gate strategy (C). Isotype controls (in red) have been used to analyze the expression of markers (blue). Representative plots are shown in each cell type. Viab: viability. [file Image_1.tif]

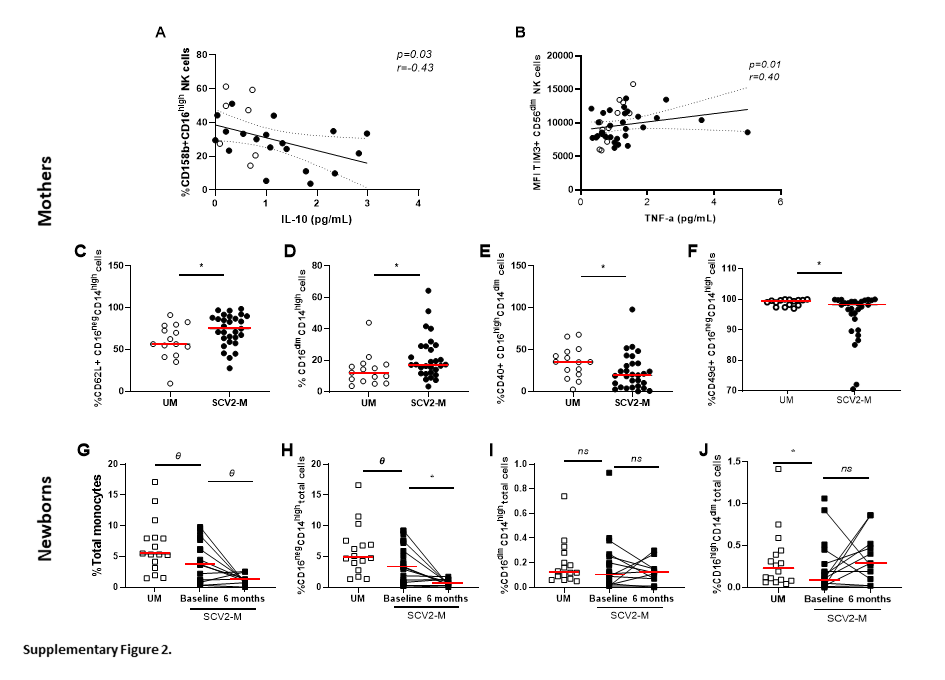

Supplement: Supplementary Figure 2 — NK cells correlations with cytokine levels at baseline and phenotype of monocytes subsets. Correlation of CD158b in CD16high NK cells and soluble IL-10 (A); correlation of median-intensity fluorescence (MFI) TIM-3 in CD56dim NK cells and soluble TNF-α (B). CD62L expression in classical monocytes (CD16neg CD14high), intermediate monocytes proportion (CD16dim CD14high) and CD40 expression in patrolling monocytes (CD16high CD14dim) (C-E); CD49 expression in classical monocytes (F); frequency of total monocytes (CD14+HLA-DR+) and classical, intermediate and patrolling monocytes cell subsets in newborns (G-J). Mann-Whitney U-test was used to compare groups. Wilcoxon test was conducted to compare paired events. The Spearman ρ correlation coefficient test was used. SCV2-M are highlighted with black dots and white dots represent UM. SCV2-M, SARS-CoV2 mothers’ group; UM, Uninfected mothers’ group. ** p ≤ 0.01, * p<0.05, Ɵ 0.05≤p ≤ 0.1, ns p>0.1 [file Image_2.tif]

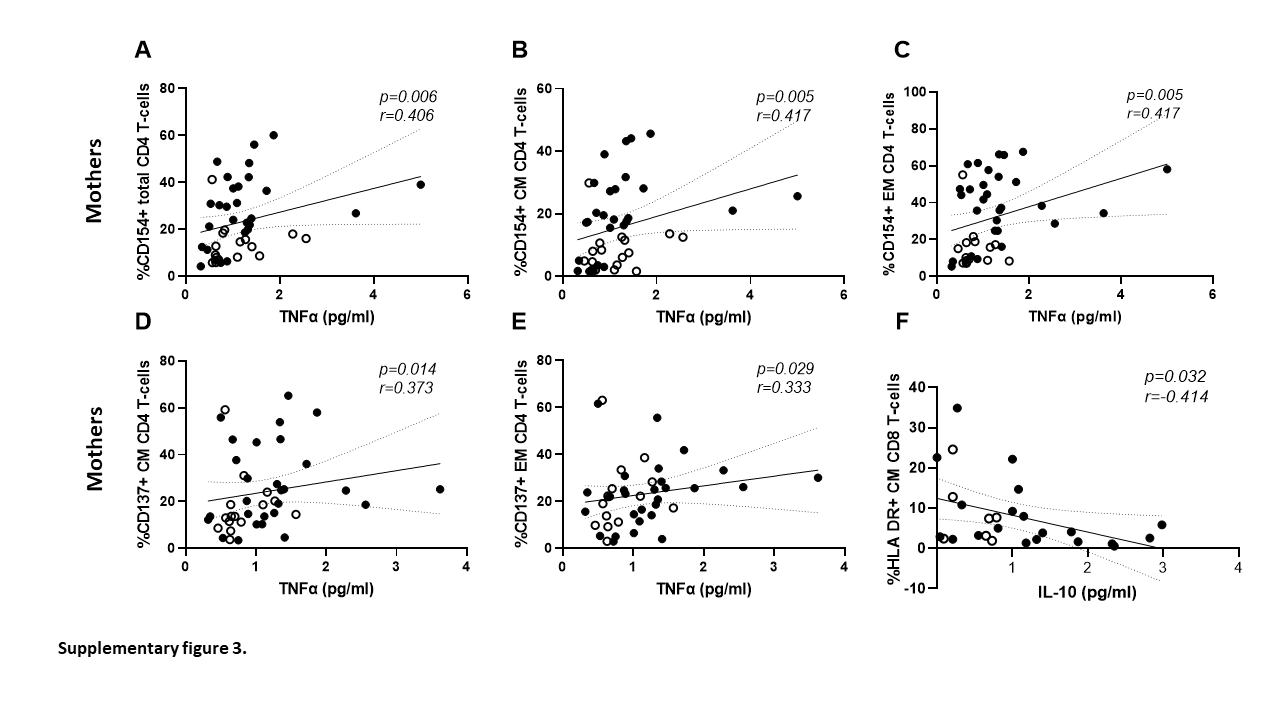

Supplement: Supplementary Figure 3 — Correlations between soluble cytokine levels and activation markers on T-cells from all mothers at baseline. Correlation of CD154 and CD137 markers in total, Central and Effector Memory (CM and EM, respectively) CD4 T-cell subsets with soluble TNF-α (A-E). Correlations of HLA-DR markers in CM CD8 T-cell subset with soluble IL-10 levels (F). IL-10 data were only available from 24 mothers. SCV2-M are highlighted with black dots and white dots represent UM. Values are taken at baseline. The Spearman ρ correlation coefficient test was used. [file Image_3.tif]

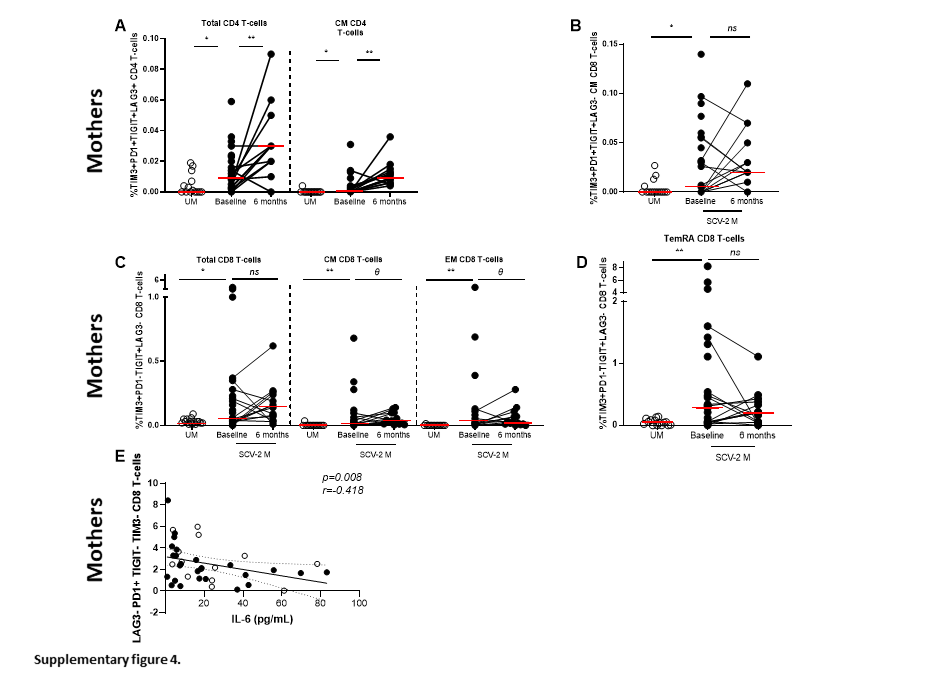

Supplement: Supplementary Figure 4 — Multiple exhaustion phenotype on T-cells at baseline and 6 months later and association between exhaustion phenotype on CD8 T-cells and soluble cytokine levels at baseline. Simultaneous expression of four exhaustion markers (TIM3+PD1+TIGIT+LAG3+) in total and Central Memory (CM) CD4 T-cell subsets (A). Combination of three exhaustion markers (TIM3+PD1+TIGIT+LAG3-) in CM CD8 T-cells (B). Expression of three exhaustion markers combination (TIM3+PD1-TIGIT+LAG3-) in total CD8-T cells and Central, Effector and Terminally Differentiated Memory subsets (CM, EM and TemRA) (C, D). Correlation between exhaustion markers (LAG3-PD1+TIGIT-TIM3-) in total CD8 T-cells and soluble IL-6 (E). Mann-Whitney U-test was used to compare groups. Wilcoxon test was conducted to compare paired events. The Spearman ρ correlation coefficient test was used. SCV2-M are highlighted with black dots and white dots represent UM. SCV2-M, SARS-CoV2 mothers’ group; UM, Uninfected mothers’ group. **p ≤ 0.01, *p<0.05, Ɵ 0.05≤p ≤ 0.1, ns p>0.1 [file Image_4.tif]
