# Supplementary material for: SARS-CoV2 Infection During Pregnancy Causes Persistent Immune Abnormalities in Women Without Affecting the Newborns
Source: Front Immunol. 2022 Jul 14;13:947549. doi: 10.3389/fimmu.2022.947549 (PMC9330630; doi:10.3389/fimmu.2022.947549)
Supplement: Supplementary file 5 [file DataSheet_1.docx]

**Supplementary Table 1. Description of antibodies markers and Isotypes controls included in T-cells, NK cells and monocytes panels analyzed by multiparametric flow cytometry.**

| **PANEL** | **Clone** | **Reference**  **And**  **Trading house** | **Isotype** | **Reference**  **And**  **Trading house** |
| --- | --- | --- | --- | --- |
| **T-cell Exhaustion** | | | | |
| LIVE/DEAD fixable Aqua Blue Dead Cell Stain |  | L34966  Life Technologies |  |  |
| CD56-BV510 | NCAM16.2 | 563041  BD Biosciences |  |  |
| CD19-BV510 | SJ25C1 | 562947  BD Biosciences |  |  |
| CD14-BV510 | MOP9 | 563079  BD Biosciences |  |  |
| CD3-APC-Cy7 | SK7 | 557832  BD Biosciences |  |  |
| CD4-APC-R700 | RPA-T4 | 564975  BD Biosciences |  |  |
| CD45RA-ECD | 2H4 | B49193  Beckman Coulter |  |  |
| CD27-PerCP-Cy5.5 | M-T271 | 560612  BD Biosciences |  |  |
| CD57-FITC | NC1 | B49188  Beckman Coulter |  |  |
| TIM-3-PE | 7D3 | 563422  BD Biosciences | Ms IgG1,k PE | 981804  Biolegend |
| PD1-BV421 | EH12.1 | 562516  BD Biosciences | Ms IgG1,k BV421 | 400158  Biolegend |
| LAG-3-Pe-Cy7 | 7H2C65 | 369310  Biolegend | Ms IgG1,k PE-Cy7 | 400126  Biolegend |
| TIGIT-AF647 | A15153G | 372724  Biolegend | Ms IgG2a,k AF647 | 400234  Biolegend |
| **T-cell Activation** | | | | |
| LIVE/DEAD fixable Aqua Blue Dead Cell Stain |  | L34966  Life Technologies |  |  |
| CD56-BV510 | NCAM16.2 | 563041  BD Biosciences |  |  |
| CD19-BV510 | SJ25C1 | 562947  BD Biosciences |  |  |
| CD14-BV510 | MOP9 | 563079  BD Biosciences |  |  |
| CD3-APC-Cy7 | SK7 | 557832  BD Biosciences |  |  |
| CD8-PB | RPA-T8 | 344718  Biolegend |  |  |
| CD27-PerCP-Cy5.5 | M-T271 | 560612  BD Biosciences |  |  |
| HLA-DR-APC | GRB-1 | HLADRA-100T  Beckman Coulter |  |  |
| CD154-PeCy7 | 24-31 | 310832  Biolegend | Ms IgG1,k PE-Cy7 | 400126  Biolegend |
| CD137-PE | 4B4-1 | 555956  BD Biosciences | Ms IgG1,k PE | 981804  Biolegend |
| CD38-FITC | HB7 | 555459  BD Biosciences |  |  |
| **Treg** | | | | |
| LIVE/DEAD fixable Aqua Blue Dead Cell Stain |  | L34966  Life Technologies |  |  |
| CD3-PerCP-Cy5.5 | SK7 | 332771  BD Biosciences |  |  |
| CD4-APC-Cy7 | OKT4 | 317418  Biolegend |  |  |
| CD31-AF647 | WM59 | 561654  BD Biosciences | Ms IgG1,k AF647 | 400130  Biolegend |
| CD127-PeCy7 | R34.34 | A64618  Beckman Coulter |  |  |
| CD25-BV421 | 9F10 | 302630  Biolegend | Ms IgG1,k BV421 | 400158  Biolegend |
| FoxP3-PE | 259D/C7 | 12-4776-42  BD Biosciences | Ms IgG1,k PE | 981804  Biolegend |
| **NK cells** | | | | |
| LIVE/DEAD fixable Aqua Blue Dead Cell Stain |  | L34966  Life Technologies |  |  |
| CD3-BV510 | SK7 | 564713  BD Biosciences |  |  |
| CD14-BV510 | MOP9 | 563079  BD Biosciences |  |  |
| CD19-BV510 | SJ25C1 | 562947  BD Biosciences |  |  |
| CD56-APC-Cy7 | NCAM16.2 | 318332  Biolegend |  |  |
| CD16-PerCP-Cy5.5 | 3G8 | 560717  BD Biosciences |  |  |
| CD57-FITC | NC1 | B49188  Beckman Coulter |  |  |
| TIM-3-PE | 7D3 | 563422  BD Biosciences | Ms IgG1,k PE | 981804  Biolegend |
| HLA-DR-APC | GRB-1 | HLADRA-100T  Beckman Coulter |  |  |
| CD158b-PE-Cy7 | DX27 | 339512  Biolegend | Ms IgG1,k PE-Cy7 | 400126  Biolegend |
| NKG2D-PECF594 | 1D11 | 562498  BD Biosciences | Ms IgG1,k PECF594 | 562292  BD Biosciences |
| NKG2A-BV421 | 131411 | 747924  BD Biosciences | Ms IgG2a,k BV421 | 400260  Biolegend |
| **Monocytes** | | | | |
| LIVE/DEAD fixable Aqua Blue Dead Cell Stain |  | L34966  Life Technologies |  |  |
| CD56-BV510 | NCAM16.2 | 563041  BD Biosciences |  |  |
| CD19-BV510 | SJ25C1 | 562947  BD Biosciences |  |  |
| CD3-BV510 | SK7 | 564713  BD Biosciences |  |  |
| HLA-DR-APC | GRB-1 | HLADRA-100T  Beckman Coulter |  |  |
| CD14-APC/Cy7 | MφPg | 557831  BD Biosciences |  |  |
| CD16-PerCP-Cy5.5 | 3G8 | 560717  BD Biosciences |  |  |
| CD62L-PECF594 | DREG-56 | 562301  BD Biosciences | Ms IgG1,k PECF594 | 562292  BD Biosciences |
| CD49d-BV421 | 9F10 | 304322  Biolegend | Ms IgG1,k BV421 | 400158  Biolegend |
| CD163-FITC | GHI-61 | 563697  BD Biosciences | Ms IgG1,k FITC | 400110  Biolegend |
| CD40-PE/Cy7 | 5C3 | 561215  BD Biosciences | Ms IgG1,k PE-Cy7 | 400126  Biolegend |
| CD287-PE | 4G6 | MA5-16249  Life Technologies | Ms IgG1,k PE | 981804  Biolegend |

**Supplementary Table 2. Frequency of total NKs, monocytes, T cells and subset distribution in SARS-CoV2 exposed newborns at baseline and 6 months later and non-exposed newborns.**

|  | **UM** | **SCV2+** | | ***p-value*** | |
| --- | --- | --- | --- | --- | --- |
|  |  | **Baseline** | **6 months** | ***p***  **(UM**  ***vs***  **Baseline)** | ***p* (Baseline *vs***  **6months)** |
| **T-cells** | | | | | |
| **%CD4+ total cells** | 16[3-26] | 9[0-28] | 26[7-32] | **0.02** | **<0.01** |
| **%CD31+CD4 T-cells** | 92[78-95] | 91[87-99] | 89[83-91] | 0.43 | **<0.01** |
| **%CD45RA+CD4 T-cells** | 93[87-96] | 93[89-98] | 86[77-93] | 0.20 | **<0.01** |
| **%CD31+CD45RA+CD4 T-cells** | 88[74-92] | 88[83-98] | 85[74-89] | 0.30 | **<0.01** |
| **%CD8+ total cells** | 0.2[0-0.6] | 0.2[0-5] | 0.5[0-1] | 0.91 | **<0.01** |
| **%CD31+CD8 T-cells** | 93[89-97] | 94[83-100] | 86[64-96] | 0.20 | **<0.01** |
| **%CD45RA+CD8 T-cells** | 89[82-96] | 92[73-100] | 81[53-91] | 0.12 | **<0.01** |
| **%CD31+CD45RA+CD8 T-cells** | 84[79-95] | 89[64-100] | 74[52-86] | **0.02** | **<0.01** |
| **%CD4+CD8+ total cells** | 0.2[0-0.6] | 0.1[0-1] | 0.5[0-1] | 0.53 | **<0.01** |
| **NK cells** | | | | | |
| **%CD56^dim^ total cells** | 15[6-31] | 10[1-25] | 9[2-21] | **0.03** | 0.70 |
| **%CD56^high^ total cells** | 1[0.6-2] | 1[0-2] | 1[0-2] | **0.01** | 0.31 |
| **% CD16^high^ total cells** | 8[2-15] | 4[0-17] | 9[1-12] | 0.05 | 0.70 |
| **%CD56^dim^ NK cells** | 99[94-100] | 99[89-100] | 99[96-100] | 0.06 | 0.21 |
| **%CD56^high^ NK cells** | 8[4-19] | 9[4-27] | 10[3-21] | 0.99 | 0.16 |
| **%CD16^high^ NK cells** | 48[31-73] | 51[8-70] | 65[48-82] | 0.87 | **0.03** |
| **Monocytes** | | | | | |
| **%Total monocytes** | 5[2-17] | 4[0-10] | 1[0-3] | 0.06 | 0.08 |
| **%CD16^neg^CD14^high^ total cells** | 5[1-16] | 3[0-9] | 0.5[0-2] | 0.05 | **0.02** |
| **%CD16^neg^CD14^high^ monocyte cells** | 89[78-96] | 90[49-97] | 52[32-96] | 0.93 | **0.01** |
| **%CD16^dim^CD14^high^ total cells** | 0.1[0-1] | 0.1[0-1] | 0.1[0-0.3] | 0.15 | 0.65 |
| **%CD16^dim^CD14^high^ monocyte cells** | 4[1-5] | 3[1-15] | 11[1-22] | 0.45 | **0.01** |
| **%CD16^high^CD14^dim^ total cells** | 0.2[0-1] | 0.1[0-1] | 0.3[0-0.8] | **0.03** | 0.24 |
| **%CD16^high^CD14^dim^ monocyte cells** | 6[0-14] | 3[0-20] | 33[1-61] | 0.5 | **<0.01** |

Values are taken at baseline and 6 months later. Continuous variables are expressed as the medians and interquartile ranges [IQR]. Mann-Whitney U-test was used to compare groups. Wilcoxon test was conducted to compare paired events. The frequency of total cells has been calculated with respect to the pool of live PMBCs. SCV2-M, SARS-CoV2 mothers’ group; UM, Uninfected mothers’ group. P-values <0.05 are highlighted.
